# Modeling household transmission dynamics: Application to waterborne diarrheal disease in Central Africa

**DOI:** 10.1371/journal.pone.0206418

**Published:** 2018-11-07

**Authors:** Casper Woroszyło, Boseung Choi, Jessica Healy Profitós, Jiyoung Lee, Rebecca Garabed, Grzegorz A. Rempala

**Affiliations:** 1 Mathematical Biosciences Institute, The Ohio State University, Columbus, 43210 Ohio, United States of America; 2 Department of National Statistics, Korea University, Sejong, 30019, Republic of Korea; 3 Division of Environmental Health Sciences, College of Public Health, The Ohio State University, Columbus, 43210 Ohio, United States of America; 4 Department of Food Science and Technology, The Ohio State University, Columbus, 43210 Ohio, United States of America; 5 Department of Veterinary Preventive Medicine, The Ohio State University, Columbus, 43210 Ohio, United States of America; 6 Division of Biostatistics, College of Public Health, The Ohio State University, Columbus, 43210 Ohio, United States of America; Public Library of Science, UNITED KINGDOM

## Abstract

**Introduction:**

We describe a method for analyzing the within-household network dynamics of a disease transmission. We apply it to analyze the occurrences of endemic diarrheal disease in Cameroon, Central Africa based on observational, cross-sectional data available from household health surveys.

**Methods:**

To analyze the data, we apply formalism of the dynamic SID (susceptible-infected-diseased) process that describes the disease steady-state while adjusting for the household age-structure and environment contamination, such as water contamination. The SID transmission rates are estimated via MCMC method with the help of the so-called synthetic likelihood approach.

**Results:**

The SID model is fitted to a dataset on diarrhea occurrence from 63 households in Cameroon. We show that the model allows for quantification of the effects of drinking water contamination on both transmission and recovery rates for household diarrheal disease occurrence as well as for estimation of the rate of silent (unobserved) infections.

**Conclusions:**

The new estimation method appears capable of genuinely capturing the complex dynamics of disease transmission across various human, animal and environmental compartments at the household level. Our approach is quite general and can be used in other epidemiological settings where it is desirable to fit transmission rates using cross-sectional data.

**Software sharing:**

The R-scripts for carrying out the computational analysis described in the paper are available at https://github.com/cbskust/SID.

## Introduction

Diarrhea often occurs as a symptom of an infection in the intestinal tract caused by a bacterial, viral or parasitic organism. Such infections are typically spread through drinking water, contaminated food, or from animal-to-person and from person-to-person as a result of poor hygiene [1, 2]. Most people who die from diarrheal diseases actually die from severe dehydration and fluid loss. Children who are malnourished or have impaired immunity as well as people living with HIV are most at risk of life-threatening diarrhea. Indeed, diarrhea is one of the primary killers of the young children worldwide, with an estimated 1.7 billion annual cases of diarrhea among children under 5 resulting in over 500,000 deaths, the majority occurring in low and middle income countries [3].

Although diarrheal disease is common across all economic settings, it has the most potential to cause severe consequences when resources and medical care are limited or when co-morbidities are present. Acute episodes of disease more quickly lead to dangerous dehydration, while chronic gastrointestinal infection is now thought to be linked to environmental enteric disorder (EED), which results in a chronically damaged gut, reduced immunity, and stunted growth [4]. Loss of linear growth, particularly in a child’s first years of life, can have long lasting impacts on cognitive and motor development [5]. However, consequences of disease aside, it remains unclear how differences in exposures and susceptibility play a role in the overall difference observed between children’s and adults’ diarrhea incidence.

In looking at household exposures to pathogens that may cause diarrhea, it appears that the interaction with animals (whether pets, livestock, or wildlife) plays an important role [3, 4, 6]. However, the potential of childhood animal exposure to modulate immunity and allergies [7–10] and of livestock ownership to improve nutrition and economic stability for families [4, 6] means that the specific role of household animals in transmission of diarrheal disease is complicated and needs to be clarified and better quantified with the help of a more mechanistic model.

The investigation of mechanisms behind household transmission of pathogens that cause diarrhea is not easy due to the complexity of the disease and its persistent endemic state in the global human population [3]. In general, the disease may be caused by both human-specific as well as zoonotic pathogens that have a variety of life cycles and the sheer number of potential culprits makes determining the specific cause of all observed cases on any sort of large scale practically impossible [3, 11]. In many developing countries (including most of Africa, see [12]) the problem is additionally compounded by the fact that most of the health surveillance programs operate with limited resources, and the data to assess transmission of diarrhea is generally limited to demographics, reported incidence of diarrhea, and possibly some outcome measures on households or individuals testing positive for a particular pathogen [11, 13]. Although such data may be used with the traditional mechanistic models to ascertain the role of different pathogens and transmission pathways on incidence of diarrhea [14, 15], the traditional models have difficulty adjusting for the presence of unrelated, endemic baseline of diarrhea occurrences [16, 17].

### Our contribution

**Fig 1:**
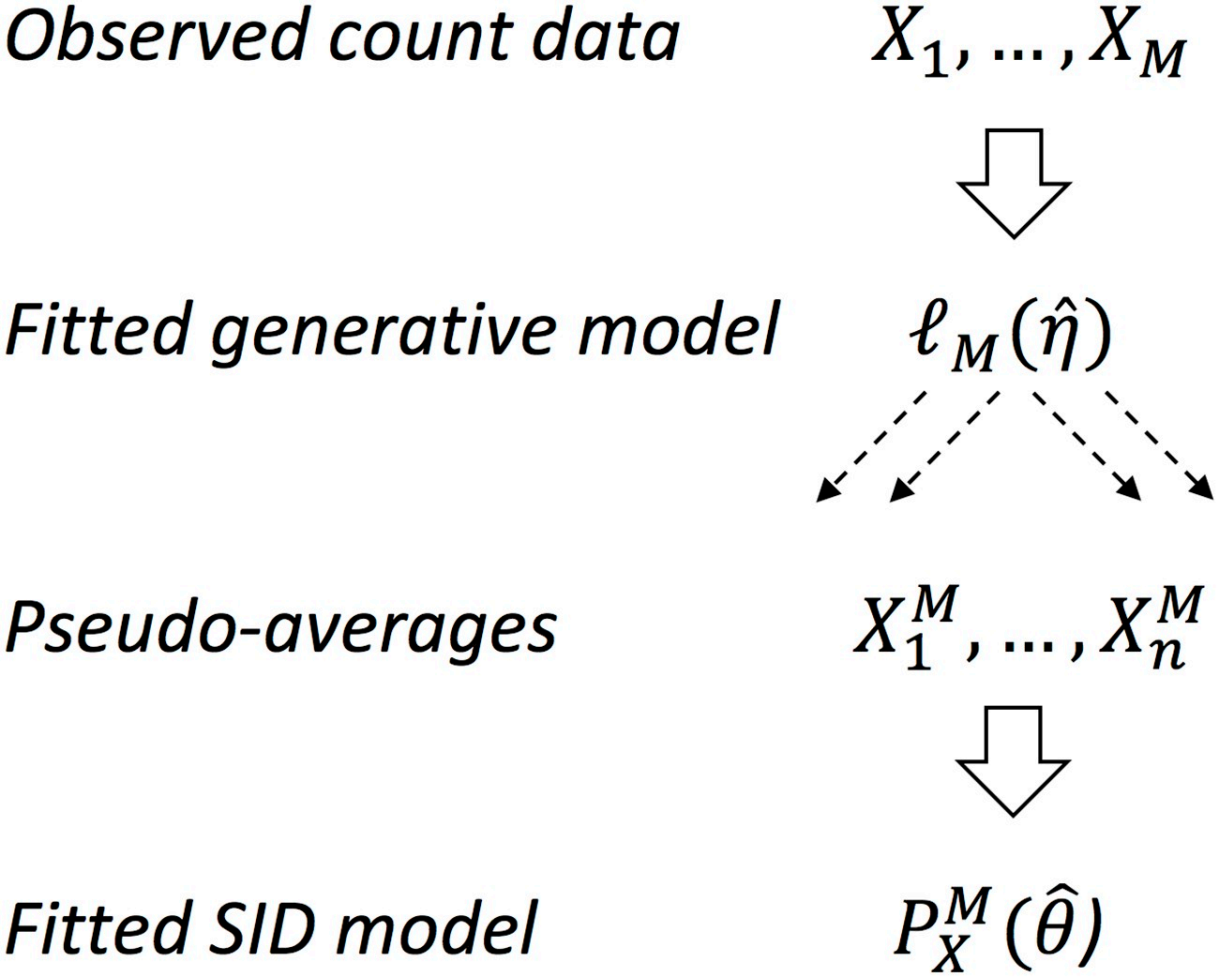
Synthetic inference based on some data *X*_*obs*_ and the SID likelihood $P_{X}^{M}(\hat{\theta }$). The count data *X*_1_, … *X*_*M*_ represents household level diarrhea cases among adults and juveniles under and contaminated (*V* = 1) and clean (*V* = 0) environments and is used to fit the generative model (observed likelihood) $\ell_{M}\left(\hat{\eta }\right)$ based on (1). The generated pseudo-data $X_{1}^{M},\ldots X_{n}^{M}$ are then used to fit the SID model $P_{X}^{M}\left(\hat{\theta }\right)$ based on (2) and (3).

In this paper we propose a method of modeling transmission pathways of diarrhea using symptoms occurrence data from individual households consisting of family members with different susceptibility (for instance, children and adults) in the environment subject to waterborne pathogen contamination and possibly also other risks effecting baseline incidence rates. Our approach is quite general and allows to adjust not only for these different causes of diarrhea, even with data of poor resolution, but also for a variety of confounders typically encountered in similar observational studies. The particularly relevant confounders in our setting are the cases of non-symptomatic infectives and uninfected symptomatics. An example of a dataset of interest, as obtained from a cross-sectional study of Cameroon households, is presented in Table 1. The dataset is especially interesting as it matches the results of household drinking water testing for pathogens with detailed household health survey and demographic data. For the type of observational data in Table 1 we propose to first fit the household-level occurrence model and then to apply parametric resampling technique akin to *the synthetic likelihood* (see, e.g., [18, 19]) to obtain approximate distribution of the mean occurrence across households. Due to some general approximation results for a wide class of counting processes (see [20] chapter 11) we may assume here that the mean of the diarrhea occurrence is well-approximated by the stationary state of a certain system of ordinary differential equations (ODEs) with additive normal noise. This main idea behind our proposed approach is summarized in Fig 1.

**Table 1:** Example of several data records from the dataset of *M* = 63 Maroua households. Full dataset provided in S1 Data.

Household Id	Water Contamination	Adults Sympt/Total	Juveniles Sympt/Total
1	No	0/2	0/0
4	Yes	0/6	0/4
24	Yes	1/4	0/2
51	No	2/2	0/0

Note that without the intermediate resampling step it is in general not possible to obtain the estimates of the transmission dynamics simply from cross-sectional occurrence. However, under the assumption of a constant risk (which is typically tacitly made in similar studies) we may consider the observed cases of diarrhea as a statistical sample from a stationary disease process. In this case, the ODEs parameters may be identified using the Bayesian inference techniques with the help of an MCMC algorithm (see, e.g., [21]). Using this approach we are able to obtain all relevant posterior estimates, including transmission rates and the expected count of latent infections (infection present but no diarrhea symptoms) as well as disease-unrelated occurrences (diarrhea symptoms present but no infection). Details of the analysis method are provided in the next section. To our knowledge, ours is the first application of the synthetic likelihood/resampling method to observational data on household diarrhea occurrence. We hope that similar approaches can be applied to larger datasets and consequently help improve current guidelines for treatment and intervention for diarrhea [2].

## Materials and methods

### Occurrence data and observed likelihood

To perform our analysis we use data from the observational study investigating the relationship between household drinking water quality and diarrhea occurrence in Maroua, Cameroon. The data was described in [22] and more recently in [12]. Briefly, the study examined the relation between the occurrences of diarrhea and the presence of gastrointestinal pathogens within home drinking water sources in four urban neighborhoods in Maroua, the regional capital of northern Cameroon. For the purpose of the study diarrhea was defined as three or more loose bowel movements (“selles molles” in French) per day.

Heads of household assented to participation in the study with the use of a verbal consent script. In addition, other members of the household present for the survey assented to the survey and water sampling. Assent was recorded through use of a verbal consent script by the technicians collecting samples and administering the survey. The protocol was approved by the Ohio State University Institutional Review Board/ Human Research Protection Program (Federal-wide Assurance #00006378 from the Office for Human Research Protections in the Department of Health and Human Services: protocol 2010B0004). Within this ethical review for the survey the protocol was approved for a waiver of signed consent forms due to the low literacy of the population and cultural inappropriateness of obtaining signatures to record consent.

Diarrhea occurrence data and water samples from home water storage containers were collected from *M* = 63 households. Pathogen contamination was assessed using qPCR method, targeting several potential zoonotic pathogens including *Campylobacter* spp., Shiga toxin producing *Escherichia* coli (*stx*1 and *stx*2), and *Salmonella* spp. Microbial source tracking (MST) targeted three different host-specific markers: HF183 (human), Rum2Bac (ruminant) and GFD (poultry) to identify fecal contamination sources. For the purpose of our analysis below the pathogen/MST levels in each household were encoded as binary outcomes (water contamination present/absent) and combined with collected demographic information on the number of household members, their age and the history of diarrhea symptoms within last 14 days. Two neighborhoods tested positive for most pathogens/MST while the others only tested positive for one or two. As *E.coli* was found in all water samples, it was excluded from our contamination criterion. Spatial variation of pathogens/MST existed between sources, storage containers, and neighborhoods but was not included in the set of covariates for current analysis due to small sample sizes of different spatial patterns. Differing population density and ethno-economic characteristics could potentially explain and correct for the variation but for the sake of simplicity we have not performed such analysis here. For illustration, several data points from the Cameroon dataset are listed in Table 1 where the diarrhea occurrences are recorded separately for adult and juvenile (under 15 years old) household members. The total number of adults and juveniles in the water contaminated (resp. uncontaminated) households was *N*_*J*_(1) = 103 and *N*_*A*_(1) = 111 (resp. *N*_*J*_(0) = 99 and *N*_*A*_(0) = 155).

Assuming that the data in Table 1 constitutes a sample from the cross-sections of a stationary distribution, each datapoint may be represented as a pair of occurrences of diarrhea (*D*_*J*_, *D*_*A*_) observed, respectively, in adult and juvenile compartments of random size (*N*_*J*_, *N*_*A*_). Because the mean and variance for the juvenile and adult compartments are approximately the same, the independent Poisson distributions are assumed for their respective sizes. Given the compartment sizes and the status of water contamination, the respective numbers of occurrences within compartments are assumed to follow binomial distributions with probabilities *p*_*J*_(*V*) and *p*_*A*_(*V*) where *V* ∈ {0, 1} denotes the presence or absence of the water contamination. Although we do not model it explicitly due to small sample sizes, we tacitly assume the functional relationship between *p*_*J*_(*V*) and *p*_*A*_(*V*). In summary, for the compartments of sizes *N*_*J*_, *N*_*A*_, and the number of symptomatic (diseased) individuals denoted by *D*_*J*_, *D*_*A*_, and the household contamination status *V*, we assume the following *generative model* for the data

$$\begin{array}{c} N_{J}∼\text{Poisson}\left(\lambda_{J}\right);D_{J}∼\text{Binomial}\left(N_{J},p_{J}\left(V\right)\right)\\ N_{A}∼\text{Poisson}\left(\lambda_{A}\right);D_{A}∼\text{Binomial}\left(N_{A},p_{A}\left(V\right)\right). \end{array}$$ (1)

Under the above model the likelihood-based inference may be now performed to estimate the compartment- and contamination-specific vector of parameters *η*_*V*_ = (*p*_*J*_(*V*), *p*_*A*_(*V*), *λ*_*J*_, *λ*_*A*_) for *V* ∈ {0, 1}. For ease of notation, in what follows we suppress the subscript *V* when describing the parameters. Further details are provided in S1 Appendix. The numerical values of the estimated parameters are given in the next section.

### SID model and synthetic likelihood

The data in Table 1 is cross-sectional and cannot be immediately used to analyze the within-household transmission pattern. Nevertheless, the generative representation via (1) allows for valid statistical inference indirectly, using the idea of synthetic likelihood akin to that proposed in [18]. Note that if we consider the sample from (1) as a set of independent realizations of some stationary counting process, then, by a version of the central limit theorem, we could expect its mean to approximately follow the normal distribution centered at a stationary solution of a certain ODE system (see [23] chapter 5). For the particular problem in hand, it is natural to take the ODE system to be one describing a compartmental SID (susceptible-infected-diseased) model defined below. Accordingly, the fitted generative model (1) may be used to generate *n* independent batches of *M* pseudo-data (denoted *X*_*obs*_, see below) with corresponding *n* averages (denoted $X_{obs}^{M}$, see below) following a normal distribution with mean determined by the stationary SID system of ODEs.

**Fig 2:**
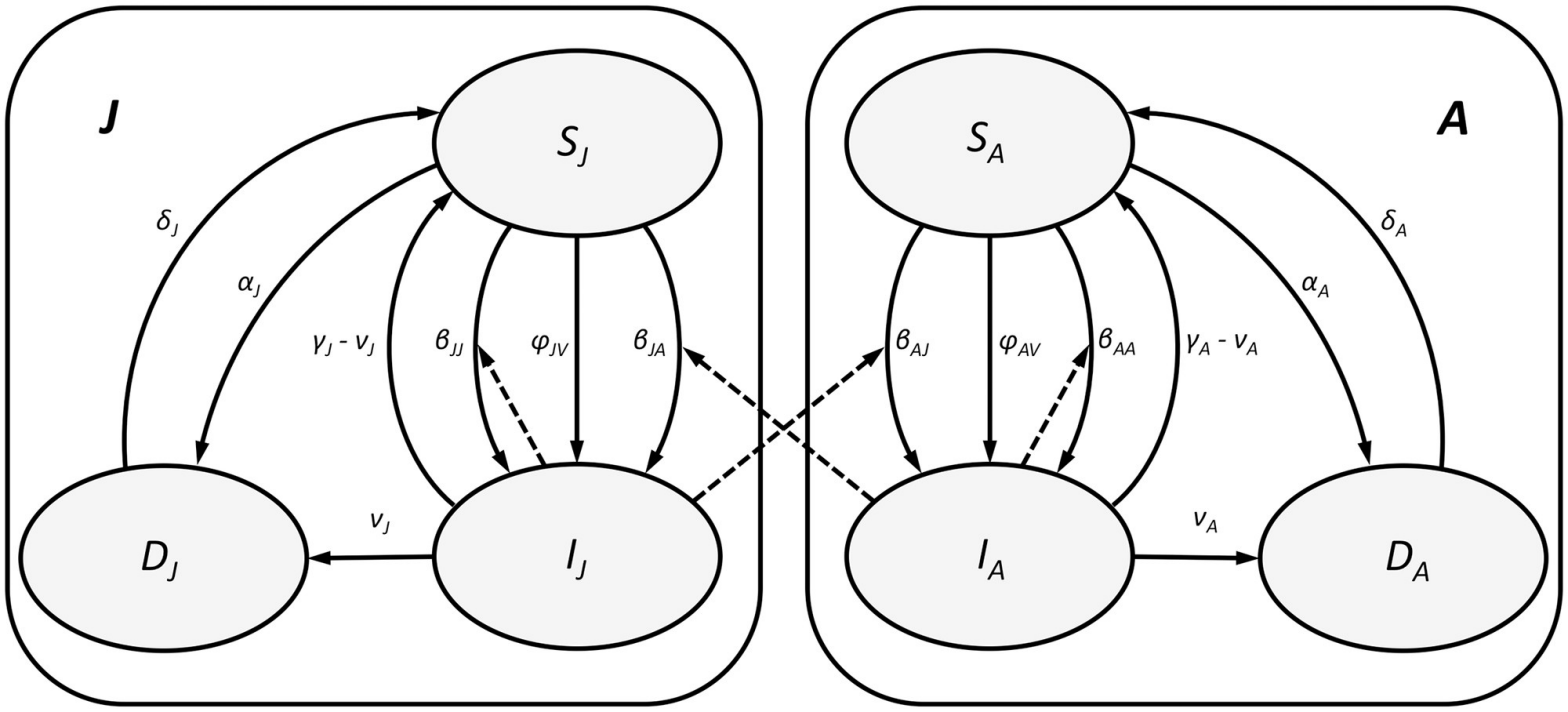
The graphical representation of the SID model from Table 2 with marked two compartments *J* (juveniles) and *A* (adults). Solid lines denote transitions within compartments. Dashed lines indicate transitions due to interactions (both within and across compartments) between susceptible (S) and infected (I) individuals.

In order to describe the SID model and introduce the required notation, denote the household-observed number of non-symptomatic and non-infected, adults (resp. juveniles) by *S*_*A*_ (resp. *S*_*J*_), the non-symptomatic but already pathogen infected adults (resp. juveniles) by *I*_*A*_ (resp. *I*_*J*_), and the symptomatic, or diseased, either infected or non-infected, adults (resp. juveniles) by *D*_*A*_ (resp. *D*_*J*_). The complete data for a given household with environment *V* ∈ {0, 1} comprises the vector *X* = (*S*_*J*_, *I*_*J*_, *D*_*J*_, *S*_*A*_, *I*_*A*_, *D*_*A*_, *V*) although in practice (due to lack of symptoms among *I*’s) only the vector *X*_*obs*_ with the aggregated counts of the non-symptomatic ${\tilde{D}}_{A}=S_{A}+I_{A}$ and ${\tilde{D}}_{J}=S_{J}+I_{J}$ as well as *D*_*A*_, *D*_*J*_ and *V* is observable. Under these assumptions, the Maroua data (cf. Table 1) may be considered as the set of *M* = 63 independent observations of the random vector *X*_*obs*_. We denote the empirical mean of *X*_*obs*_ based on *M* observations by $X_{obs}^{M}$ and assume that it follows the normal distribution with mean given by the stationary compartmental SID model, as summarized in Table 2 and Fig 2. Since in the actual dataset only a single vector $X_{obs}^{M}$ is available, we generate additional means vectors from the *pseudo-data* using (1) as described above. As seen in Table 2, depending on the status of contamination (*V* ∈ {0, 1}), our SID model is parametrized by the vector ***θ***_*V*_ of 12 (*V* = 0) or 14 (*V* = 1) parameters. As before, we suppress the subscript *V* in what follows and write

$$\begin{array}{c} \theta =(\beta_{JJ},\beta_{JA},V\phi_{J},\gamma_{J},\beta_{AA},\beta_{AJ},V\phi_{A},\gamma_{A},\alpha_{J},\nu_{J},\delta_{J},\alpha_{A},\nu_{A},\delta_{A}) \end{array}$$

to denote the appropriate rates of transmission and recovery/infection between different model compartments and types.

**Table 2:** The reaction network description of the SID model with two compartments (*i*, *j* ∈ {*A*, *J*}). The graphical representation of the network is provided in Fig 2 and the corresponding ODE system in (A.2) in S1 Appendix.

Rate Parameter	Transition	Rate Parameter	Transition
*β*_*ij*_	(*S*_*i*_, *I*_*j*_) → (*I*_*i*_, *I*_*j*_)	*Vϕ*_*i*_	*S*_*i*_ → *I*_*i*_
*α*_*i*_	*S*_*i*_ → *D*_*i*_	*δ*_*i*_	*D*_*i*_ → *S*_*i*_
*ν*_*i*_	*I*_*i*_ → *D*_*i*_	*γ*_*i*_ − *ν*_*i*_	*I*_*i*_ → *S*_*i*_

As summarized in Table 2, for *i*, *j* ∈ {*A*, *J*}, *β*_*ij*_ denotes the rate at which $S_{i}^{M}$, through interaction with $I_{j}^{M}$, converts into $I_{i}^{M}$; *Vϕ*_*i*_ denotes the rate at which infected environment (*V* = 1) converts $S_{i}^{M}$ into $I_{i}^{M}$ (*Vϕ*_*i*_ = 0 for *V* = 0); *α*_*i*_ denotes the rate at which $S_{i}^{M}$ converts into $D_{i}^{M}$ and *δ*_*i*_ is the rate of the reverse conversion. Finally, *ν*_*i*_ denotes the rate at which $I_{i}^{M}$ progresses to $D_{i}^{M}$ and *γ*_*i*_ − *ν*_*i*_ denotes the rate at which $I_{i}^{M}$ returns back to $S_{i}^{M}$. The graphical diagram of all the transitions and interactions in Table 2 is presented in Fig 2.

The corresponding ODE system describing the SID dynamics is presented in (A.2) in S1 Appendix. Based on that system we may relate the generated pseudo-data to model parameters as follows. Consider the average number of household asymptomatic individuals in adult and juvenile groups and denote

$$\begin{array}{c} {\tilde{D}}_{A}^{M}≔S_{A}^{M}+I_{A}^{M}\;\text{and}\;{\tilde{D}}_{J}^{M}≔S_{J}^{M}+I_{J}^{M}. \end{array}$$

Solving the SID model ODE for its steady state we obtain, on one hand,

$$\begin{array}{cc} {\tilde{D}}_{J}^{M}= & (\frac{\gamma_{J}}{\beta_{JJ}I_{J}+\beta_{JA}I_{A}+V\phi_{J}}+1)I_{J}≕f_{1}^{\theta_{1}}\left(I_{J},I_{A}\right)\\ {\tilde{D}}_{A}^{M}= & (\frac{\gamma_{A}}{\beta_{AA}I_{A}+\beta_{AJ}I_{J}+V\phi_{A}}+1)I_{A}≕f_{2}^{\theta_{2}}\left(I_{J},I_{A}\right) \end{array}$$ (2)

and, on the other,

$$\begin{array}{cc} {\tilde{D}}_{J}^{M}= & \frac{\left(\alpha_{J}-\nu_{J}\right)I_{J}+\delta_{J}D_{J}}{\alpha_{J}}≕f_{3}^{\theta_{3}}\left(I_{J}\right)\\ {\tilde{D}}_{A}^{M}= & \frac{\left(\alpha_{A}-\nu_{A}\right)I_{A}+\delta_{A}D_{A}}{\alpha_{A}}≕f_{4}^{\theta_{4}}\left(I_{A}\right). \end{array}$$ (3)

where the *f*’s are defined by their left-hand sides and we denote ***θ***_1_ = (*β*_*JJ*_, *β*_*JA*_, *Vϕ*_*J*_, *γ*_*J*_), ***θ***_2_ = (*β*_*AA*_, *β*_*AJ*_, *Vϕ*_*A*_, *γ*_*A*_), ***θ***_3_ = (*α*_*J*_, *ν*_*J*_, *δ*_*J*_), and ***θ***_4_ = (*α*_*A*_, *ν*_*A*_, *δ*_*A*_), so that ***θ*** = (***θ***_1_, ***θ***_2_, ***θ***_3_, ***θ***_4_).

Note that the quantities ${\tilde{D}}_{A}^{M}$ and ${\tilde{D}}_{J}^{M}$ are derived from the pseudo-data $X_{obs}^{M}$ obtained by sampling from the fitted model (1).

### Parameter estimation

Due to a relatively small size *M* of the dataset, we do not attempt to evaluate the variable *V* dynamically but instead consider two separate SID models for contaminated and uncontaminated environments (*V* = 1 and *V* = 0). In each case, in order to estimate the vector of parameters ***θ*** as well as two hidden states (*I*_*A*_, *I*_*J*_) based on the generated sample of *n* pseudo-averages $X_{obs}^{M}$, we employ an MCMC procedure. Its advantage is in being able to handle the latent (unobservable) variables and in providing a simple and intuitive way of validating the final model against observations in Table 1. The disadvantage is in a relatively high computational overhead due to a somewhat complicated Metropolis-within-Gibbs algorithm [24] described in Algorithm 1 below. Details on the forms of the conditional distributions are provided in S1 Appendix. To ease notation, let ***θ***_−*k*_ denote the vector ***θ*** with its *k*-th component removed (*k* = 1, …, 4). Recall that when *V* = 0 the *ϕ* parameter is 0 and hence is excluded from from ***θ***_1_ and ***θ***_2_. We estimate parameters ***θ*** separately for *V* = 0, 1 via the following iterative procedure.

#### MCMC algorithm for SID model fitting

1. Given the state of environment *V* ∈ {0, 1} generate a collection ${\tilde{d}}_{V}\left(n\right)$ of *n* pseudo-data points $\left({\tilde{d}}_{i}^{A},{\tilde{d}}_{i}^{J}\right)\left(V\right)$, each of them being an average of *M* independent draws of the pair $\left({\tilde{D}}_{A},{\tilde{D}}_{J}\right)\left(V\right)$ from (1) under fitted parameters $\hat{\eta }$.
2. Initiate values of the rate vector ***θ*** as well as *I*_*A*_(*V*), and *I*_*J*_(*V*), according to their prior distributions (see S1 Appendix).
3. Using the Metropolis-Hastings (MH) step, conditionally on (*I*_*A*_, *I*_*J*_)(*V*) and ${\tilde{d}}_{V}\left(n\right)$, draw sequentially samples from the conditional distributions of ***θ***_*k*_|***θ***_−*k*_, *k* = 1, …, 4. The form of the proposal in MH step as well as the forms of the conditionals are given in (A.4)–(A.7) in S1 Appendix
4. Using the MH step, conditionally on ***θ*** and ${\tilde{d}}_{V}\left(n\right)$, draw independently from *I*_*A*_(*V*) and *I*_*J*_(*V*) using their conditionals as given in (A.8) and (A.9) in S1 Appendix.
5. Repeat step 3 and 4 until convergence.

In our analysis, we iterated the above MCMC procedure 40,000 times retaining every 10-th iteration for *V* = 0 and 20-th iteration for *V* = 1, in order to ensure good chain mixing. We also removed the first 20,000 iterations as a burn-in set and summarized the posterior statistics based on the remaining iterations. To check for the robustness of our analysis with respect to the amount *n* of the generated pseudo-data, we applied the MCMC algorithm above with *n* = 50 and *n* = 100, however, since the results were virtually identical, we only report below on the case *n* = 100. Although larger values of *n* could be also considered, this particular value seems to strike a good balance between required MCMC precision and computational overhead.

#### Model validation

The final step in our model estimation procedure was validation against the observed data. This was done by comparing the posterior distributions of the model generated data samples using estimated parameters with the actually observed values from *X*_*obs*_ and looking for large departures from the posterior mode.

#### Software

The R-scripts for carrying out tour computational analysis described above along with the Maroua dataset adapted from [22] are available at https://github.com/cbskust/SID.

## Results

The initial set of fitted parameters obtained for the generative model (1) based on the *M* = 63 Maroua households dataset is provided in Table 3. As can be seen from the entries in the table, an interesting feature of this dataset appears to be that the probability of diarrhea in the juvenile compartment is *decreased* in the households with contaminated water environment (*V* = 1). There may be several reasons for this finding which appears inconsistent with other reported observational studies [25]. First, the survey data for juveniles may be less reliable than for adults, particularly in young children who under our definition are also a part of the juvenile compartment. Second, it is known [26] that a substantial number of juvenile diarrhea cases is, in fact, unrelated to the waterborne infections and the collected data may be simply confounded with this unrelated process. Finally, it is also possible that the contaminated environment offers some measure of immunity from diarrhea, perhaps due to non-specific activation of the immune system [26].

**Table 3:** Estimates in data generating model based on the observed likelihood (1). Estimates of *λ* are pooled across *V* values.

Water Contamination (*V*)	*p*_*J*_	*p*_*A*_	*λ*_*J*_	*λ*_*A*_
Yes (*V* = 1)	0.1262	0.1261	3.2063	4.2222
No (*V* = 0)	0.1414	0.0710	3.2063	4.2222

The numerical results of the MCMC-based fitting of ***θ*** for SID model under both *V* = 0 and *V* = 1 are summarized in Table 4 where we list the posterior means, posterior standard deviations, and 95% credible intervals (CIs) based on the generated *n* = 100 pseudo-data points and 2000 thinned posterior samples. Complementing the table entries, the full sets of marginal densities and trace plots for the posterior distributions are provided, in S1–S4 Figs of the Supporting Information.

**Table 4:** Summary of MCMC results based on *n* = 100 pseudo-households.

	Water contaminated (*V* = 1)			Water clean (*V* = 0)		
	Mean	Std Dev	95% CI	Mean	Std Dev	95% CI
*β*_*JJ*_	**0.4921**	0.4212	(0.0296, 1.5966)	**0.5275**	0.4293	(0.0392, 1.6873)
*β*_*JA*_	0.4950	0.4224	(0.0348, 1.5245)	0.4677	0.3962	(0.0355, 1.5137)
*ϕ*_*J*_	0.5159	0.4227	(0.0233, 1.6213)			
*γ*_*J*_	0.7700	0.5357	(0.0761, 2.1018)	0.7104	0.4933	(0.0795, 2.0047)
*β*_*AA*_	0.4829	0.3699	(0.0404, 1.3745)	0.4563	0.3699	(0.0317, 1.4145)
*β*_*AJ*_	**0.5562**	0.4443	(0.0298, 1.6465)	**0.4748**	0.3771	(0.0269, 1.4428)
*ϕ*_*A*_	0.5318	0.4228	(0.0561, 1.7337)			
*γ*_*A*_	0.7847	0.5688	(0.092, 2.3176)	0.8219	0.5571	(0.0999, 2.1469)
*α*_*J*_	**0.4892**	0.4824	(0.0651, 1.7999)	**0.7266**	0.4694	(0.1424, 1.8301)
*ν*_*J*_	**0.1415**	0.1409	(0.0131, 0.5821)	**0.2318**	0.1980	(0.0157, 0.7836)
*δ*_*J*_	0.9223	0.4685	(0.1324, 1.9421)	0.7711	0.5515	(0.0599, 2.2047)
*α*_*A*_	**0.7269**	0.5331	(0.1042, 2.1272)	**0.8243**	0.5424	(0.1194, 2.2096)
*ν*_*A*_	**0.1927**	0.1728	(0.0144, 0.6356)	**0.2192**	0.1952	(0.0168, 0.7247)
*δ*_*A*_	**0.7880**	0.5270	(0.0972, 1.9188)	**0.6314**	0.4654	(0.0605, 1.8727)
$I_{J}^{M}$	2.2634	1.0453	(0.2444, 3.7829)	1.6109	0.4300	(0.6082, 2.2321)
$I_{A}^{M}$	**3.3334**	0.7008	(1.4657, 4.1644)	**3.0352**	0.6349	(1.4452, 3.7943)

Although we opted not to conduct the direct comparison of the parameter values in ***θ*** between *V* = 0 and *V* = 1, one may somewhat informally perform such a comparison based on the CI entries in Table 4. In general, if for a particular parameter in ***θ*** its CI bounds under *V* = 0 are contained within the CI bounds under *V* = 1, or vice-versa, one would consider the corresponding posterior distributions as statistically (i.e., for given data) equal. To facilitate such analysis in Table 4 the parameters with statistical distinct posterior distributions are entered in bold. From the entries in Table 4 it therefore follows that although the posterior distributions of the transmission rates *β*_*JJ*_ and *β*_*AJ*_ are statistically different between *V* = 0 and *V* = 1, it is not so for the remaining rates *β*_*AA*_ and *β*_*JA*_. Similarly, we find that although the average number of silent infections among juveniles under *V* = 0 and *V* = 1 (mean $I_{J}^{M}\left(1\right)=2.2634$ vs mean $I_{J}^{M}\left(0\right)=1.6109$) is not statistically different, this is not the case for the average number of silent infections among adults, despite the smaller absolute difference of their means. (This particular finding appears to be due to the relatively large value of the posterior standard deviation of *I*_*J*_(1).) Similar comparisons may be also performed between the recovery rates. Indeed, we find that while the recovery rate in the adult compartment is significantly slowed down in the contaminated environment (mean *δ*_*A*_(1) = 0.7880 vs mean *δ*_*A*_(0) = 0.6314), the rate in the juvenile compartment is not significantly changed.

From the view point of waterborne disease, the most interesting are perhaps the estimates of the water contamination effects on the households diarrhea persistence in different compartments. In Table 4 our SID model quantifies the effect of water contamination (*V* = 1) in the households on average as *ϕ*_*A*_ = 0.5318 and *ϕ*_*J*_ = 0.5159. This indicates that despite the differences in the diarrhea prevalence patterns among juveniles and adults (see Table 3), the overall effect of waterborne pathogens is quantitatively similar. Note that the simple estimates in Table 3 which are based on the survey data (Table 1) and ignore the SID dynamics and asymptomatic infections suggest otherwise (cf. also [12]). Note also that according to the SID model the average number of infectious individuals (both pre-symptomatic and never-symptomatic) is larger in the contaminated environment, with the observed difference being significant in the adult compartment. Moreover, Eqs (2) and (3) for the specific values in Table 4 indicate that remediating contaminated water environment in the household (moving from *V* = 1 to *V* = 0) is likely to remove the symptomatic cases in the average adult compartment but not so in the juvenile one. Finally, let us also note that the observed higher prevalence of diarrhea among juveniles Table 3 in the clean environment may be explained on the basis of our SID model by the higher transmission in the juvenile compartment (*β*_*JJ*_(0) is significantly greater then *β*_*JJ*_(1)) and an increase in non-pathogen/ non-household diarrhea (increased *α*_*J*_).

**Fig 3:**
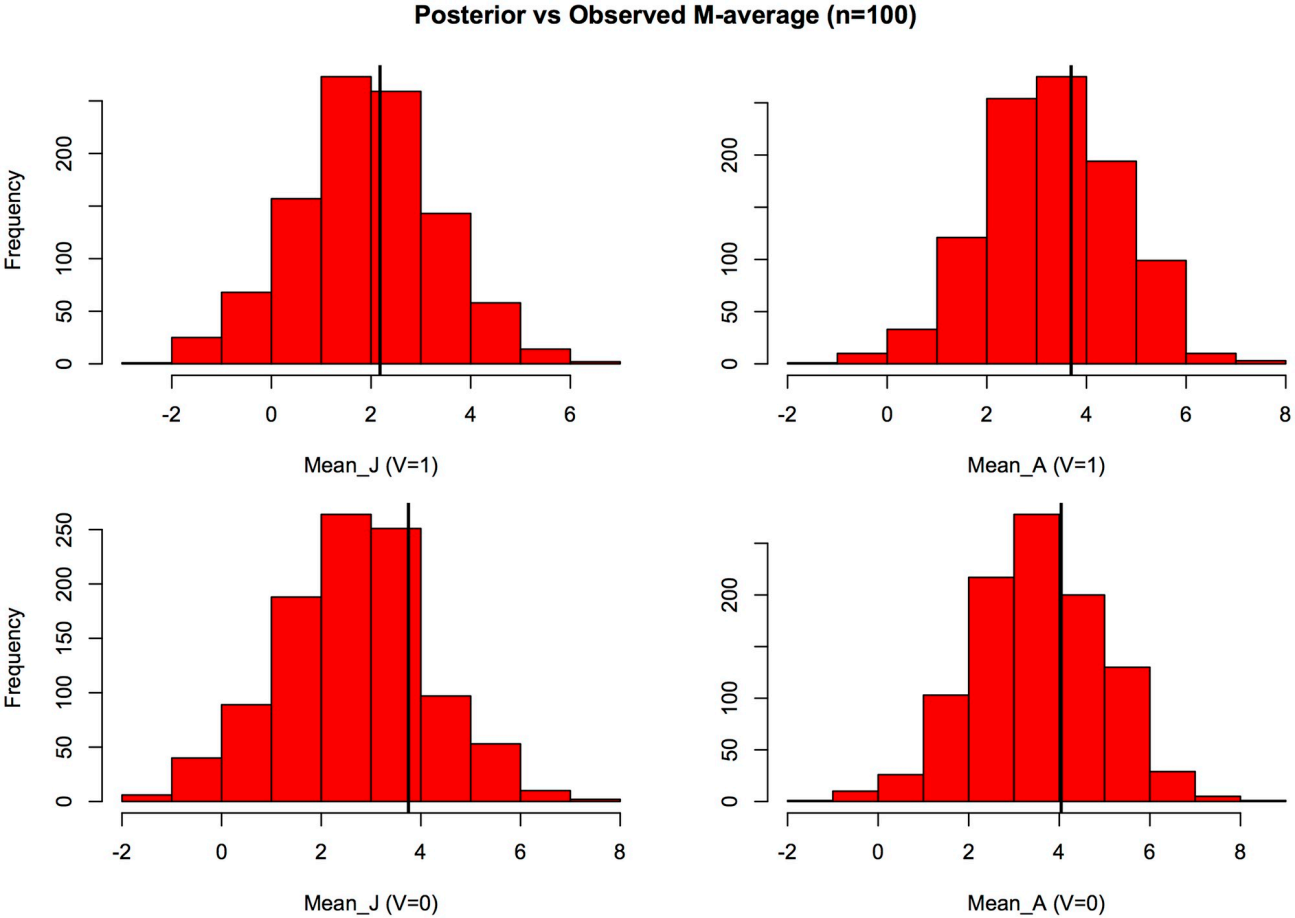
Model validation. The distributions of the posterior means of the counts of asymptomatic individuals in juvenile (J) and adult compartments based on the fitted SID model (2) and (3) vs the actual observed values from *M* = 63 Maroua households (cf. Table 1) marked by vertical lines.

The results of model validation are shown separately for the *A* and *J* compartments in Fig 3 where the numerical values of the means of *X*_*obs*_ (vertical lines) are plotted along with the corresponding histograms of their posterior distributions obtained from the model with estimated parameters. As seen from the plots, the observed values are within the reasonable range of the posterior mode and hence may be considered in agreement with the fitted model. This also implies that the CI bounds in Table 4 may be interpreted as plausible ranges of respective parameter values consistent with the observed data. These ranges are quite wide indicating somewhat large uncertainty, likely due to moderate sample size (*M* = 63).

## Summary and discussion

In many observational disease studies we lack the ability to collect repeated measurements over time, either due to cost or practicality considerations. Consequently, disease transmission studies often have to rely on cross-sectional data containing latent variables and multiple confounders (e.g. latent infections or different disease susceptibility across population). For such data we have proposed here a statistical method for direct analysis of the transmission rates across different population compartments and different environmental risk factors. The ideas for statistical analysis came from the consideration of stationary SID model based on differential equations and synthetic likelihood with MCMC algorithm for estimating parameters. The proposed estimation method appears to be quite stable and capable of converging in a relatively large parameter space (in our example we had up to 16 parameters) even when supplied with only slightly informative prior distributions for moderate sample size.

Applying modern Bayesian approach to fit SID transmission model allowed us to better account for the uncertainty of various model components (i.e., bias or lack of accuracy) as well as the uncertainty of outcomes predictions (i.e., variance or lack of precision). It also allowed us to naturally incorporate any additional information about the model parameters. For instance, should some of the estimated compartmental diarrhea probabilities be fixed at specific values (say, based on prior studies) the fitting algorithm could easily incorporate this additional information. In such case one would expect to see both model’s precision and accuracy to increase. We also note that in our example dataset the posterior marginal distributions of the parameters were all unimodal, indicating that the model parameters were identifiable, that is, their joint posterior distribution had a unique mode contained in the range of plausible parameter values given the observed data. In general, our proposed statistical approach may be viewed as an alternative to a more traditional epidemiological disease risk analysis based on the odds ratios, where the Cochran-Mantel-Haenszel (CMH) stratification method is typically used to adjust for confounders.

The example dataset we have chosen was part or a larger study investigating possible links between drinking water contamination and diarrheal diseases in urban environment of Central/Sub-Saharan Africa [12, 22]. Although this particular study did not specifically examine other factors associated with gastrointestinal infections (socioeconomic status, overall sanitation, household education, storage, etc), they likely did contribute to the observed baseline (not water-related) occurrence. However, our statistical analysis indicated that in our dataset they constituted only a small minority of the observed symptomatic cases.

In order to better appreciate the possible implications of SID-type analysis for public health policies and interventions, it is helpful to compare its results (Table 4) with the results from initial, purely descriptive analysis of the Maroua dataset (Table 3) akin to that conducted previously in [22]. We note that since descriptive analysis in Table 3 is based on risks comparison (i.e., the binomial probabilities) it provides only an aggregated measure of the water contamination effect on the prevalence of diarrhea. It is not clear in particular what specific transmission pathways should be targeted for intervention in order to minimize the observed occurrence (note that the juvenile risk is actually smaller in contaminated households). In contrast, the SID analysis in Table 4 provides (via Eqs (2) and (3)) an explicit numerical relations between transition rates and occurrence, and therefore a comprehensive picture of competing household transmission risks. Consequently, the SID analysis allows for a more detailed examination of how household occurrence risk is associated with the water environment and how it is transferred across age compartments. Such information appears essential for developing more targeted water intervention strategies beyond those that are currently recommended by WHO (see, [2] Section 11.3) for reducing diarrhea risk.

## Supporting information

S1 Fig Marginal plots for the posterior parameters of the SID model under *V* = 1 and *n* = 100 with 2,000 iterations. (TIF)

S2 Fig Marginal plots for the posterior parameters of the SID model under *V* = 0 and *n* = 100 with 2,000 iterations. (TIF)

S3 Fig Diagnostic trace plots for the posterior parameters of the SID model under *V* = 1 and *n* = 100 with 2,000 iterations. (TIF)

S4 Fig Diagnostic trace plots for the posterior parameters of the SID model under *V* = 0 and *n* = 100 with 2,000 iterations. (TIF)

S1 Appendix Appendix on statistical analysis. Contains additional formulas and derivations related to the statistical analysis.
(PDF)

S1 Data Maroua household data. Diarrhea symptoms data from 63 households from Maroua, Cameroon. The household ID, number of juveniles (J) and adults (A) as well as the number of respective symptomatics (DJ and DA).
(CSV)
