# Supplementary figures and images for: Modeling household transmission dynamics: Application to waterborne diarrheal disease in Central Africa
Source: PLoS One. 2018 Nov 7;13(11):e0206418. doi: 10.1371/journal.pone.0206418 (PMC6221320; doi:10.1371/journal.pone.0206418)

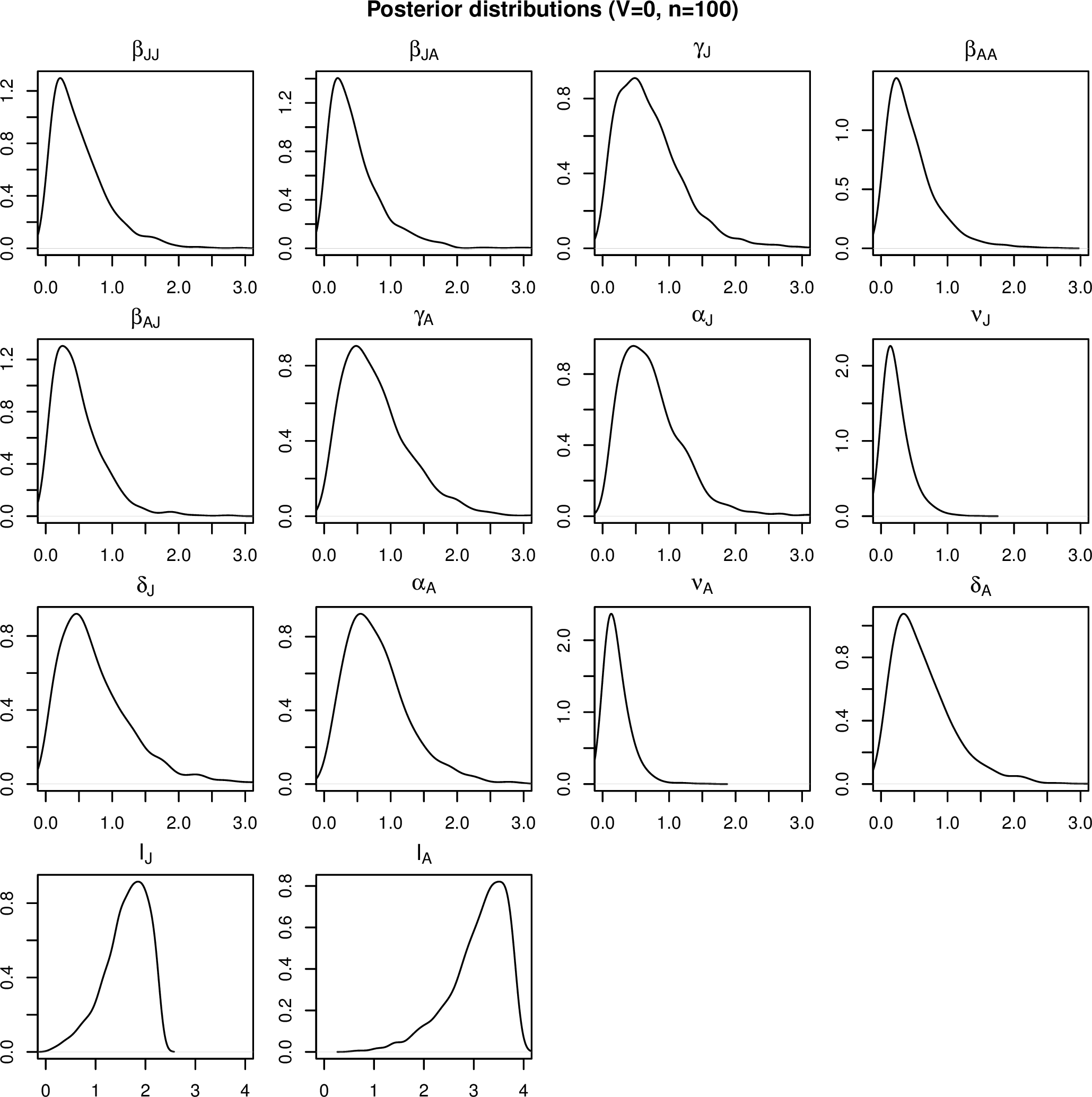

Supplement: S1 Fig — (TIF) [file pone.0206418.s001.tif]

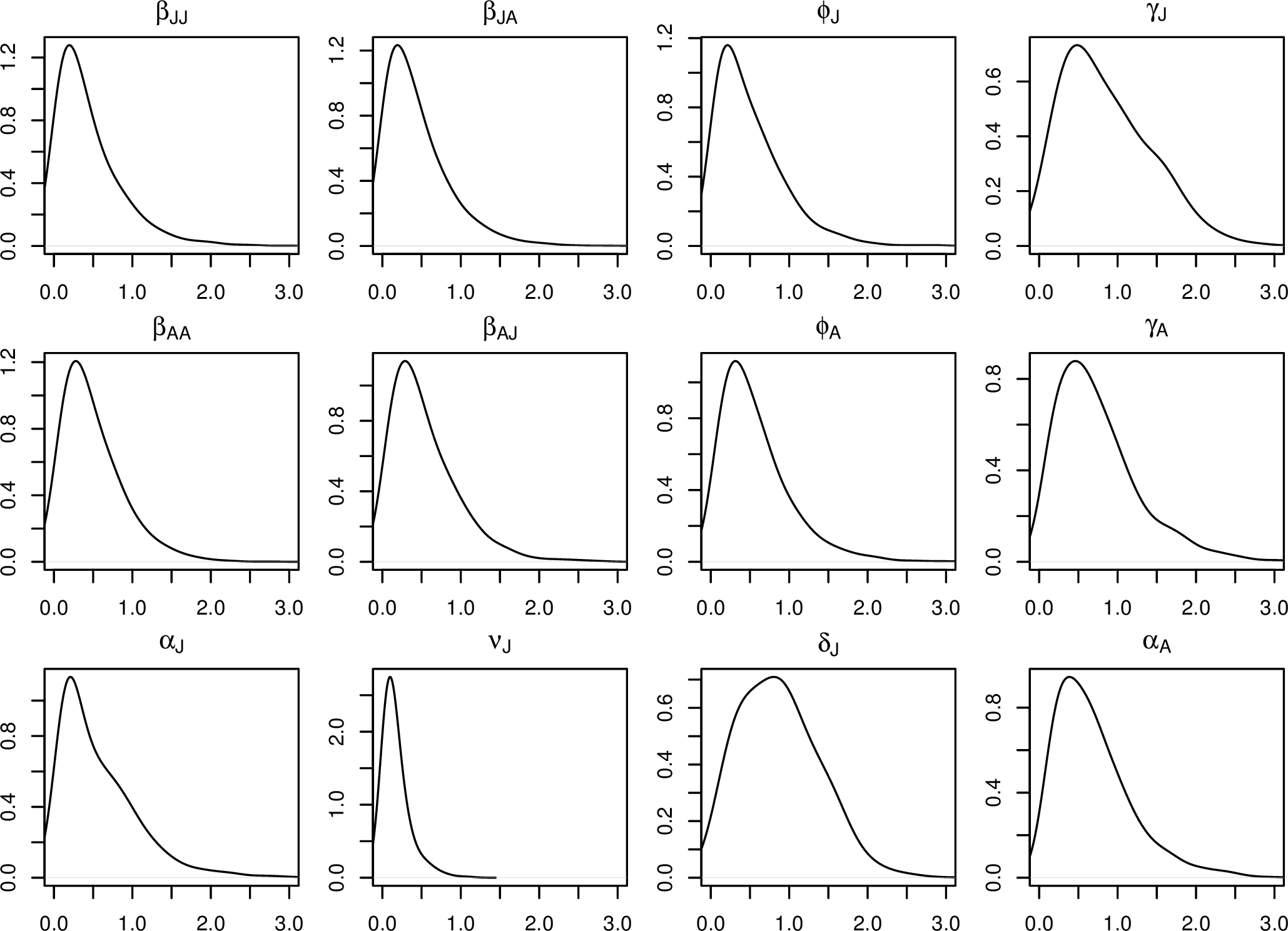

Supplement: S2 Fig — (TIF) [file pone.0206418.s002.tif]

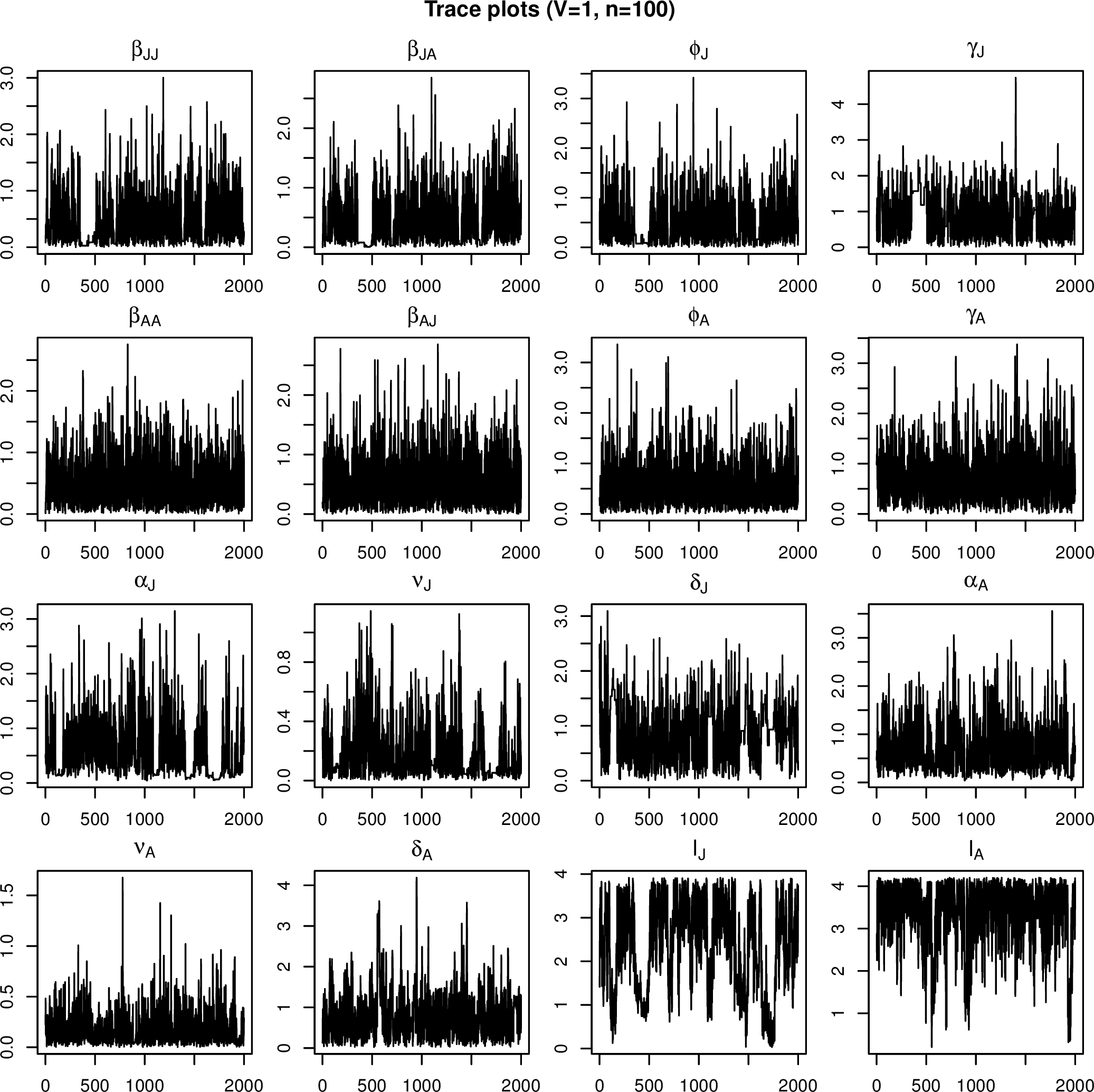

Supplement: S3 Fig — (TIF) [file pone.0206418.s003.tif]

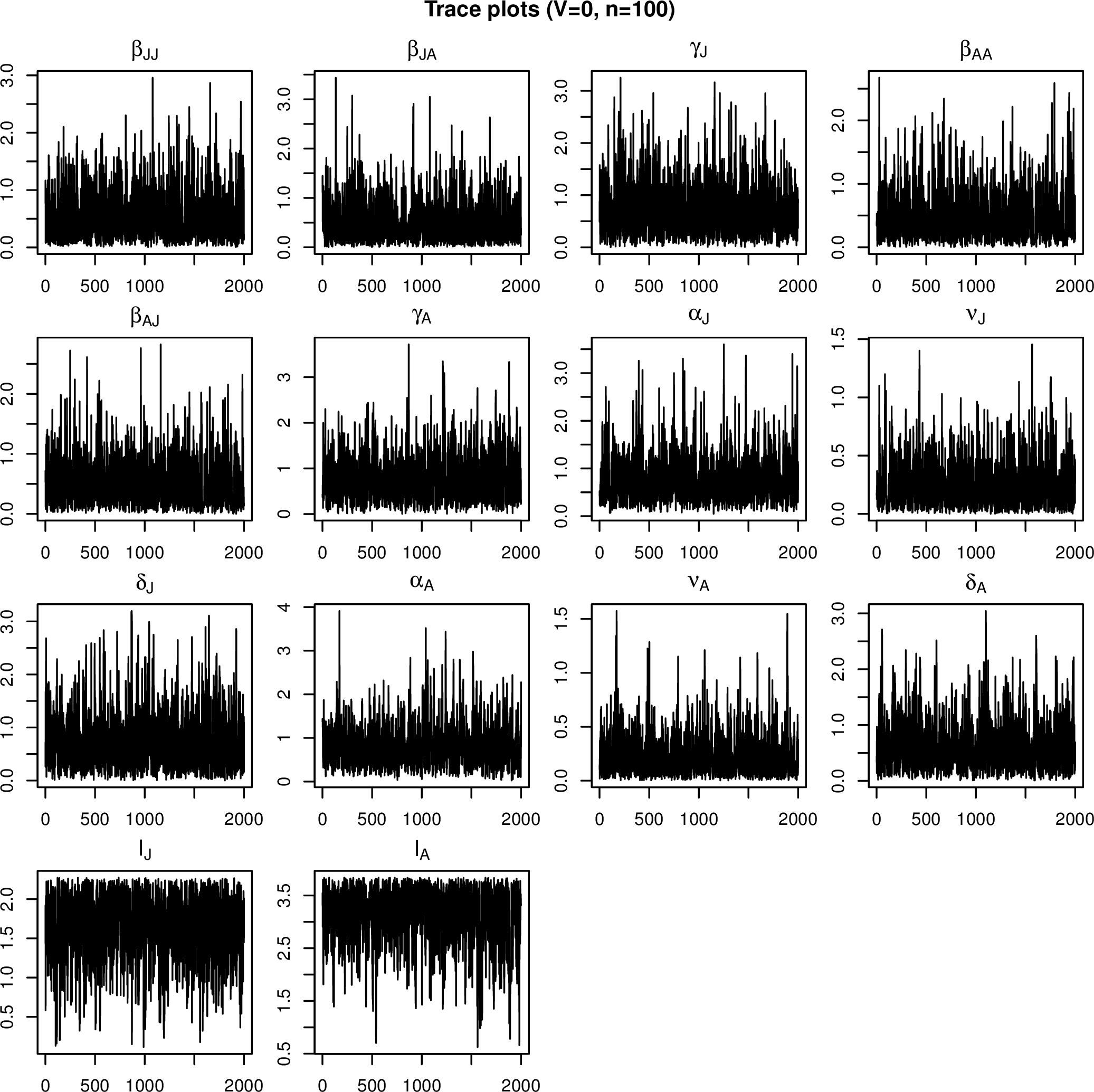

Supplement: S4 Fig — (TIF) [file pone.0206418.s004.tif]
