# Supplementary material for: Modeling household transmission dynamics: Application to waterborne diarrheal disease in Central Africa
Source: PLoS One. 2018 Nov 7;13(11):e0206418. doi: 10.1371/journal.pone.0206418 (PMC6221320; doi:10.1371/journal.pone.0206418)
Supplement: S1 Appendix — Contains additional formulas and derivations related to the statistical analysis. (PDF) [file pone.0206418.s005.pdf]

# Modeling Household Transmission Dynamics: Application to Waterborne Diarrheal Disease in Central Africa

Casper Woroszyło[1,◑], Boseung Choi[2,◑], Jessica Healy Profitós[3], Jiyoung Lee[3,4], Rebecca Garabed[5], Grzegorz A. Rempala [1,6,*]

**1** Mathematical Biosciences Institute, The Ohio State University, Columbus, 43210 Ohio, U.S.A.
**2** Department of National Statistics, Korea University, Sejong, 30019, Republic of Korea
**3** Division of Environmental Health Sciences, College of Public Health, The Ohio State University, Columbus, 43210 Ohio U.S.A.
**4** Department of Food Science and Technology, The Ohio State University, Columbus, 43210 Ohio U.S.A.
**5** Department of Veterinary Preventive Medicine, The Ohio State University, Columbus, 43210 Ohio, U.S.A.
**6** Division of Biostatistics, College of Public Health, The Ohio State University, Columbus, 43210 Ohio U.S.A.

◑These authors contributed equally to this work.
∗ Corresponding author: rempala.3@osu.edu

## APPENDIX: Additional Computational Details

**Occurrence data and observed likelihood inference.** Assuming that for each of $M$ households we have diarrhea occurrence data for two age compartments (groups) $k \in \{A, J\}$, denoted $D_{k,i}$, the total compartment size $N_{k,i}$, and the environment status $V_i$ $(i = 1, \dots, M)$. Assuming the occurrence probability is $p_k(E)$, the data generating log-likelihood $\ell_M$ as a function of the parameters vector $\eta = (p_A, p_J, \lambda_A, \lambda_J)$ and the environment $E \in \{0, 1\}$ is given by

$$\begin{aligned}\ell_M(p_A, p_J, \lambda_A, \lambda_J|E) &= \sum_{i=1}^{M}\sum_{k}\sum_{E}\left(D_{k,i}\log(p_k(V_i)) + (N_{k,i} - D_{k,i})\log(1 - p_k(V_i))\right)\mathbb{1}(V_i = E)\\ &+ \sum_{i=1}^{M}\sum_{k}\sum_{E}\left(N_{k,i}\log(\lambda_k(V_i)) - \lambda_k(V_i)\right)\mathbb{1}(V_i = E) + \mathcal{O}(D_{k,i}, N_{k,i}),\end{aligned}$$

yielding the maximum likelihood estimates

$$\begin{aligned}\hat{p}_k(E) &= \frac{\sum_{i=1}^{M} D_{k,i}\mathbb{1}(V_i = E)}{\sum_{i=1}^{M} N_{k,i}\mathbb{1}(V_i = E)}\\ \hat{\lambda}_k &= \frac{\sum_{i=1}^{M} N_{k,i}}{M}, \quad \text{for } k \in \{A, J\}. \qquad \text{(A.1)}\end{aligned}$$

**SID model and synthetic likelihood inference.** The SID model is given by the deterministic, mass-action ODE system which describes the evolution of the average number of susceptible ($S$), infected and asymptomatic ($I$) and diseased ($D$) individuals across two compartments ($A$ and $J$) as follows. Note that we have two separate models describing, respectively, contaminated and uncontaminated water supplies ($V = 1$ and $V = 0$).

$$\begin{aligned}
\frac{d}{dt}S_J &= -\beta_{JA}S_JI_A - \beta_{JJ}S_JI_J - V\phi_JS_J - \alpha_JS_J + \delta_JD_J + (\gamma_J - \nu_J)I_J \\
\frac{d}{dt}S_A &= -\beta_{AJ}S_AI_J - \beta_{AA}S_AI_A - V\phi_AS_A - \alpha_AS_A + \delta_AD_A + (\gamma_A - \nu_A)I_A \\
\frac{d}{dt}I_J &= \beta_{AJ}S_AI_J + \beta_{JJ}S_JI_J + V\phi_JS_J - \gamma_JI_J \\
\frac{d}{dt}I_A &= \beta_{JA}S_JI_A + \beta_{AA}S_AI_A + V\phi_AS_A - \gamma_AI_A \\
\frac{d}{dt}D_J &= \alpha_JS_J + \nu_JI_J - \delta_JD_J \\
\frac{d}{dt}D_A &= \alpha_AS_A + \nu_AI_A - \delta_AD_A
\end{aligned} \tag{A.2}$$

As in the main text, denoting the set of SID model rate parameters by $\boldsymbol{\theta}$, the Markov Chain Monte Carlo (MCMC) procedure is used to estimate the rate parameters $\boldsymbol{\theta}$ and the unobserved species, $I_A$ and $I_J$ separately for $V = 0$ and $V = 1$.

Recall that we denote $\boldsymbol{\theta} = (\boldsymbol{\theta}_1, \boldsymbol{\theta}_2, \boldsymbol{\theta}_3, \boldsymbol{\theta}_4)$ where $\boldsymbol{\theta}_1 = (\beta_{JJ}, \beta_{JA}, V\phi_J, \gamma_J)$, $\boldsymbol{\theta}_2 = (\beta_{AA}, \beta_{AJ}, V\phi_A, \gamma_A)$, $\boldsymbol{\theta}_3 = (\alpha_J, \nu_J, \delta_J)$, and $\boldsymbol{\theta}_4 = (\alpha_A, \nu_A, \delta_A)$. As discussed in the main text, we first generate the $n$ data points from the $M$-averages of the occurrence data from (1), denoted by ($\tilde{d}_i^J$, $\tilde{d}_i^A$, $i = 1, \ldots, n$) and treat them as the observed pseudo-data. The likelihood functions are now constructed based on the fact that the pseudo-data is approximately normally distributed with the respective mean vector given by the equations (2) and (3). Accordingly, we set

$$\begin{aligned}
l_J(\boldsymbol{\theta}) &\propto \exp\Bigg( -\sum_{i=1}^{n}(\tilde{d}_i^J - (f_1^{\boldsymbol{\theta}_1} + f_3^{\boldsymbol{\theta}_3})/2)^2/2\sigma_J^2\Bigg), \\
l_A(\boldsymbol{\theta}) &\propto \exp\Bigg( -\sum_{i=1}^{n}(\tilde{d}_i^A - (f_2^{\boldsymbol{\theta}_2} + f_4^{\boldsymbol{\theta}_4})/2)^2/2\sigma_A^2\Bigg).
\end{aligned} \tag{A.3}$$

where in the above we assign the standard deviation values based on the empirical estimates as $\sigma_J = 2.7434$ and $\sigma_A = 2.0345$. These values are also (appropriately) smaller then the assigned prior variance discussed below.

**Prior for $\boldsymbol{\theta}$.** Since all the parameters included in $\boldsymbol{\theta}_1$, $\boldsymbol{\theta}_2$, $\boldsymbol{\theta}_3$, $\boldsymbol{\theta}_4$ are rate parameters and hence should have positive values, we assign the independent gamma distribution for all 14 rate parameters with non-informative hyperparameters of 3/2 for the location and 1/3 for and scale.

**Prior for unobserved $I_J$ and $I_A$.** In the observed data set, we don't have a value for the numbers of infected $I_J$ and $I_A$. These unobserved $I_J$ and $I_A$ are treated as missing data. Hence we use a missing data imputation procedure for $I_J$ and $I_A$ during the MCMC simulation. In particular, the missing values of $I_J$ and $I_A$ act as unknown parameters to be estimated and need to be assigned suitable priors. Here use gamma priors distribution for $I_J$ and $I_A$. Since the ranges of $I_J$ and $I_A$ are $0 \leq I_J \leq \max(\tilde{d}_i^J)$ and $0 \leq I_A \leq \max(\tilde{d}_i^A)$, respectively, we select the hyper-parameters of the gammas in order for their respective 95% confidence interval to cover these ranges.

**Full conditionals for $\boldsymbol{\theta}$, $I_J$, and $I_A$.** Based on the above form of the prior distributions and the likelihood functions, the conditional posteriors $\pi(\boldsymbol{\theta}_k|\boldsymbol{\theta}_{-k}, I_J, I_A)$ $(k = 1, \ldots 4)$ as well as $\pi(I_J|\boldsymbol{\theta})$, and $\pi(I_A|\boldsymbol{\theta})$ are, respectively, proportionate to

$$\begin{aligned}&\pi(\boldsymbol{\theta}_1^*|\boldsymbol{\theta}_2, \boldsymbol{\theta}_3, \boldsymbol{\theta}_4, I_J, I_A, \tilde{d}_i^J)\\ &\propto \exp\left\{-\sum_{i=1}^{B}\left[\tilde{d}_i^J - \frac{\gamma_J^* I_J}{\beta_{JJ}^* I_J + \beta_{JA}^* I_A + V\phi_J^*} - I_J - \frac{(\alpha_J - \nu_J)I_J + \delta_J D_J}{\alpha_J}\right]^2 /2\sigma_J^2\right\}\\ &\times(\beta_{JJ}^*\beta_{JA}^* V\phi_J^*\gamma_J^*)^{a-1}\exp\{-(\beta_{JJ}^* + \beta_{JA}^* + V\phi_J^* + \gamma_J^*)b\},\end{aligned} \tag{A.4}$$

$$\begin{aligned}&\pi(\boldsymbol{\theta}_2^*|\boldsymbol{\theta}_1, \boldsymbol{\theta}_3, \boldsymbol{\theta}_4, I_J, I_A, \tilde{d}_i^A)\\ &\propto \exp\left\{-\sum_{i=1}^{B}\left[\tilde{d}_i^A - \frac{\gamma_A^* I_A}{\beta_{AJ}^* I_J + \beta_{AA}^* I_A + V\phi_A^*} - I_A - \frac{(\alpha_A - \nu_A)I_A + \delta_A D_A}{\alpha_A}\right]^2 /2\sigma_A^2\right\}\\ &\times(\beta_{AJ}^*\beta_{AA}^* V\phi_A^*\gamma_A^*)^{a-1}\exp\{-(\beta_{AJ}^* + \beta_{AA}^* + V\phi_A^* + \gamma_A^*)b\},\end{aligned} \tag{A.5}$$

$$\begin{aligned}&\pi(\boldsymbol{\theta}_3^*|\boldsymbol{\theta}_1, \boldsymbol{\theta}_2, \boldsymbol{\theta}_4, I_J, I_A, \tilde{d}_i^J)\\ &\propto \exp\left\{-\sum_{i=1}^{B}\left[\tilde{d}_i^J - \frac{\gamma_J I_J}{\beta_{JJ} I_J + \beta_{JA} I_A + V\phi_J} - I_J - \frac{(\alpha_J^* - \nu_J^*)I_J + \delta_J^* D_J}{\alpha_J^*}\right]^2 /2\sigma_J^2\right\}\\ &\times((\alpha_J^*\nu_J^*\delta_J^*)^{a-1}\exp\{-(\alpha_J^* + \nu_J^* + \delta_J^*)b\},\end{aligned} \tag{A.6}$$

$$\begin{aligned}&\pi(\boldsymbol{\theta}_4^*|\boldsymbol{\theta}_1, \boldsymbol{\theta}_2, \boldsymbol{\theta}_3, I_J, I_A, \tilde{d}_i^A)\\ &\propto \exp\left\{-\sum_{i=1}^{B}\left[\tilde{d}_i^A - \frac{\gamma_A I_A}{\beta_{AJ} I_J + \beta_{AA} I_A + V\phi_A} - I_A - \frac{(\alpha_A^* - \nu_A^*)I_A + \delta_A^* D_A}{\alpha_A^*}\right]^2 /2\sigma_A^2\right\}\\ &\times(\alpha_A^*\nu_A^*\delta_A^*)^{a-1}\exp\{-(\alpha_A^* + \nu_A^* + \delta_A^*)b\},\end{aligned} \tag{A.7}$$

$$
\begin{aligned}
&\pi(I_J^*|\boldsymbol{\theta}_1, \boldsymbol{\theta}_2, \boldsymbol{\theta}_3, \boldsymbol{\theta}_4, I_A, \tilde{d}_i^J) \\
&\propto \exp\left\{ \sum_{i=1}^{B} \left[ \tilde{d}_i^J - \frac{\gamma_J I_J^*}{\beta_{JJ} I_J^* + \beta_{JA} I_A + V\phi_J} - I_J^* - \frac{(\alpha_J - \nu_J) I_J^* + \delta_J D_J}{\alpha_J} \right]^2 / 2\sigma_J^2 \right\} \\
&\times (I_J^*)^{a_{I_J}-1} \exp\{I_J^* b_{I_J}\},
\end{aligned}
\tag{A.8}
$$

$$
\begin{aligned}
&\pi(I_A^*|\boldsymbol{\theta}_1, \boldsymbol{\theta}_2, \boldsymbol{\theta}_3, \boldsymbol{\theta}_4, I_J, \tilde{d}_i^A) \\
&\propto \exp\left\{ \sum_{i=1}^{B} \left[ \tilde{d}_i^2 - \frac{\gamma_A I_A^*}{\beta_{AJ} I_J + \beta_{AA} I_A^* + V\phi_A} - I_A^* - \frac{(\alpha_A - \nu_A) I_A^* + \delta_A D_A}{\alpha_A} \right]^2 / 2\sigma_A^2 \right\} \\
&\times (I_A^*)^{a_{I_A}-1} \exp\{I_A^* b_{I_A}\}.
\end{aligned}
\tag{A.9}
$$

**MH proposal step.** Unfortunately, the conditional distributions of (A.4)-(A.9), being the products of normal distributions and respective gamma priors, do not have closed forms. Hence, we may only sample from these conditional distributions with the help of the usual Metropolis-Hastings (MH) algorithm. The new state proposal in the MH step is generated using the multivariate normal distribution of the form

$$\boldsymbol{\theta}_k^* \sim MVN(\boldsymbol{\theta}_k^m, t_k \mathcal{I}_k), \quad \text{for } k = 1, \ldots, 4$$

where $\boldsymbol{\theta}^m$ is the current value of the sampled parameters, $\mathcal{I}_k$ is the identity matrix and the tuning constants $t_k$, $k = 1, \ldots, 4$ are selected so as to achieve an acceptance ratio of between 20% and 40%.

In a similar manner, we use a univariate normal distribution as a proposal distribution for the conditional posterior of (A.8) and (A.9). The proposal distribution has it's mean of current sample and standard deviation of $\tau_{I_J}$ and $\tau_{I_A}$ for $I_J$ and $I_A$ respectively. The $\tau_{I_J}$ and $\tau_{I_A}$ are tuning constants and are tuned so that acceptance ratio of the Metropolis-Hastings algorithm is about 44% in order to improve the chain convergence. Final diagnostic trace plots as well as the marginal plots for the posterior parameters are provided in S1 Fig – S4 Fig of Supporting Information.
